# Congestive Chill, Associated with Hæmaturia—Both Dependent upon Malarial Influence

**Published:** 1872-02

**Authors:** W. O’Daniel

**Affiliations:** Twiggs County, Ga., Treasurer of the Georgia Medical Association


(Read Before the Macon Medical Association.)

### CONGESTIVE CHILL, ASSOCIATED WITH HÆMATURIA — BOTH DEPENDENT UPON MALARIAL INFLUENCE.

BY W. O'DANIEL, M.D., TWIGGS COUNTY, GA.,

Treasurer of the Georgia Medical Association.

At ten o'clock A.M., on the 20th of December last, I was called to see Mr. S———, a young gentleman of twenty-two years, who was supposed by his parents to have had a chill on the evening before.

Upon entering the room of the patient, on account of the extreme yellow or greenish coloration of the conjunctiva and whole cutaneous surface, I thought, perhaps, he had icterus; but upon a thorough and careful examination, I found the following symptoms existing: Very great dullness of mental faculties; excruciating pain in head; pulse one hundred and thirty per minute, and weak; pupils dilated, though responded readily to light; tongue furred; skin hot and dry; great thirst; incessaet nausea and vomiting; liver and spleen enlarged, and tender upon pressure. Was informed that he voided an unusually large quantity of urine immediately after the chill passed off, which was highly colored with blood—at first red, but very soon had a very dark appearance, which led me to believe that cholæmia was present; and I am now well satisfied that there was an admixture of blood and bile in the urine. Had diarrhœa at the same time, though the discharges contained no bile that I could discover. Patient appeared to be under the influence of opiates, but upon inquiry, I was satisfied that he had *not* taken an opiate of any kind. I then came to the conclusion that the extreme dullness of mental faculties, drowsiness, and the disposition to torpor of the mental functions generally had been caused by the reabsorption of bile. I then very naturally supposed that the common duct leading to the duodenum was occluded, whereby the passage of bile into the natural channel had been entirely cut off.

When Nature's laws were thus disturbed, of course disease of the most malignant type ensued. The bile was then eliminated by the kidneys, which was plainly demonstrated by the abundance of it in the urine voided immediately after the chill passed off, and also by the want of it in the discharges while suffering with diarrhœa. It ceased to pass through the proper channel, and a great portion of it was taken up in the general circulation, thereby poisoning the whole system.

So soon as I was able to make out a diagnosis satisfactory to myself, I was satisfied that the prognosis was unfavorable, and the condition of the patient indeed precarious; but made the following prescription, which I hoped would remove the obstruction: Calomel grs. x., to be taken at once; also bi-carb. soda grs. xx., in a wine-glassful of water, whenever water was required; blister over region of liver and one on back of the neck, and large doses of quinine per anum, on account of the nausea and vomiting. Applied a solution of soda bi-carbonate all over the cutaneous surface, with the hope that a sufficiency of antacids might be absorbed, together with the internal use of them, to counteract or neutralize the supposed excess of acids in the system. Patient continued to grow worse until half-past four o'clock the same day, when he died. Was partially delirious when I first saw him, but died entirely so.

In my opinion, this disease, in its malignant form, generally terminates fatally, but I think can generally be prevented by the proper precautionary steps and the judicious use of anti-periodics, and mercurial treatment with the view of relieving the hepatic and splenitic complications which, I think, are generally present in the disease as I have described it. I also think the disease is rarely found except in malarious sections of country, and probably where repeated attacks of intermittent fever precedes this *dreadful* malady.

I think the following prescription an efficient one to break up chronic chills, which, perhaps, may very properly be called the forerunner of the above disease: ℞—Quinia ʒ ij; ferri redactum ʒ iss; ext. aconiti grs. xxx; morphiæ, strychniæ, acidi arsenicum, aa grs. iij. M., and make into sixty pills. One after breakfast and one after supper. (Or some other preparation containing arsenic, iron and quinine.)

I am well aware that a great many of the very first medical men differ from me in regard to the use of large doses of quinine in such cases, but, in my opinion, it is *the* remedy when an equalizer of the circulation is indicated. It is, as every medical gentleman knows, our great remedy to prevent the return of chills, and it is by equalizing the circulation that this is done. When the capillary circulation is equalized, there can be no congestion, and consequently no chill, as there never was a chill without congestion. It has been my favorite remedy in convulsions, in both adults and children, but more especially children, ever since I was fully satisfied as to its therapeutic action, and I can say that I have never used a remedy that has given me better satisfaction. I always give it, regardless of febrile excitement, in convulsions, especially in children. Indeed, it is one of the very best remedies we have in almost any disease in which there is a well-marked periodicity; and there is scarcely a disease to which the inhabitants of a malarious section of country are subject, that does not have a well-defined periodicity—or, as the common people would say, a good day and a bad one.

I had the pleasure and advice of my friend, Dr. Richardson, in the above case, who concurred with me in diagnosis and treatment. The Doctor has an experience of twenty years, and has never met but three cases, two of which died.

